# Identification of a systemic interferon-γ inducible antimicrobial gene signature in leprosy patients undergoing reversal reaction

**DOI:** 10.1371/journal.pntd.0007764

**Published:** 2019-10-10

**Authors:** Rosane M. B. Teles, Jing Lu, Maria Tió-Coma, Isabela M. B. Goulart, Sayera Banu, Deanna Hagge, Kidist Bobosha, Tom H. M. Ottenhoff, Matteo Pellegrini, Annemieke Geluk, Robert L. Modlin

**Affiliations:** 1 Division of Dermatology, Department of Medicine, David Geffen School of Medicine at University of California (UCLA), Los Angeles, California, United States of America; 2 Department of Molecular, Cell and Developmental Biology, University of California (UCLA), Los Angeles, California, United States of America; 3 Department of Infectious Diseases, Leiden University Medical Center, Leiden, The Netherlands; 4 National Reference Center for Sanitary Dermatology and Leprosy, Faculty of Medicine, Federal University of Uberlandia, Minas Gerais, Brazil; 5 International Center for Diarrhoeal Disease Research Bangladesh, Dhaka, Bangladesh; 6 Mycobacterial Research Laboratories, Anandaban Hospital, Kathmandu, Nepal; 7 Armauer Hansen Research Institute, Addis Ababa, Ethiopia; 8 Department of Microbiology, Immunology and Molecular Genetics, University of California (UCLA), Los Angeles, California, United States of America; George Washington University, UNITED STATES

## Abstract

Reversal reactions (RRs) in leprosy are characterized by a reduction in the number of bacilli in lesions associated with an increase in cell-mediated immunity against the intracellular bacterium *Mycobacterium leprae*, the causative pathogen of leprosy. To identify the mechanisms that contribute to cell-mediated immunity in leprosy, we measured changes in the whole blood-derived transcriptome of patients with leprosy before, during and after RR. We identified an ‘RR signature’ of 1017 genes that were upregulated at the time of the clinical diagnosis of RR. Using weighted gene correlated network analysis (WGCNA), we detected a module of 794 genes, *bisque4*, that was significantly correlated with RR, of which 434 genes were part of the RR signature. An enrichment for both IFN-γ and IFN-β downstream gene pathways was present in the RR signature as well as the RR upregulated genes in the *bisque4* module, including those encoding proteins of the guanylate binding protein (GBP) family that contributes to antimicrobial responses against mycobacteria. Specifically, *GBP1*, *GBP2*, *GBP3* and *GBP5* mRNAs were upregulated in the RR peripheral blood transcriptome, with *GBP1*, *GBP2* and *GBP5* mRNAs also upregulated in the RR disease lesion transcriptome. These data indicate that RRs involve a systemic upregulation of IFN-γ downstream genes including GBP family members as part of the host antimicrobial response against mycobacteria.

## Author summary

Reversal reaction (RR) is a major cause of tissue injury and disabilities in leprosy, resulting from the rapid onset of cell-mediated immune responses to the intracellular bacterium *Mycobacterium leprae*. To identify the mechanisms related to the increase in cell-mediated immunity in RR, we measured changes in the whole blood-derived transcriptome of patients with leprosy before, during and after RR. We identified that RRs are associated with an IFN-γ induced inflammatory response including an antimicrobial gene network containing the *GBP1*, *GBP2*, *GBP3* and *GBP5* mRNAs in the peripheral blood. Furthermore, we show that *GBP1*, *GBP2* and *GBP5* mRNAs are also upregulated at the site of disease in RR patients. In summary, our study suggests that RR involves a systemic induction of IFN-γ regulated genes that contributes to an antimicrobial response against the pathogen, releasing ligands and antigens that can further amplify the inflammatory response.

## Introduction

Leprosy is a poverty-related infectious disease caused by the intracellular pathogen *Mycobacterium leprae* that remains persistently present in pockets of developing countries causing 200,000 new cases each year, where it creates considerable health and economic burdens [1]. The disease offers a robust model of divergent immune responses correlating with the outcome of the host response to the pathogen [2]. At one end of the disease spectrum, tuberculoid leprosy (T-lep) patients typify the resistant response that restricts the growth of the pathogen, resulting in limited lesions and small bacilli numbers. At the opposite end, lepromatous leprosy (L-lep) patients represent susceptibility to disseminated infection, with numerous skin lesions and abundant bacilli. Whereas cell-mediated immunity against *M*. *leprae* is present in T-lep patients, humoral responses against the pathogen are characteristic of L-lep patients. Insights into human immune responses gained from investigations of leprosy include specific immune patterns based on cell-type- (CD4^+^ vs. CD8^+^) [3] and cytokine patterns of adaptive T cells (Th1 vs. Th2) [4–6] in host defense.

Although the polar forms of leprosy exist as chronic inflammatory states, patients can undergo a reversal reaction (RR), generally resulting in upgrading from the L-lep towards the T-lep pole with reduction or clearance of bacilli in lesions [7]. Clinically, RRs are characterized by the sudden appearance of inflammatory skin lesions characterized by erythema and edema with associated neuritis resulting in severe peripheral nerve impairment [8–10]. The tissue injury caused by the immune responses in peripheral nerves can be irreversible even with appropriate therapy, significantly increasing the morbidity and disability due to leprosy [11, 12]. These reactions can occur spontaneously at the time of clinical presentation before the initiation of treatment, representing a naturally occurring cell-mediated immune response to *M*. *leprae*. RRs also occur during the initiation of chemotherapy, leading to speculation that the breakdown of bacilli leads to induction of a cell-mediated response.

The study of patients undergoing RR provides insight into the dynamic changes associated with host defense against the invading pathogen. The immune response in RR involves an influx of CD4^+^ T cells in lesions [13], along with a shift from a Th2 to a Th1 cytokine pattern, including an increase in IFN-γ expression and a concomitant reduction in IL-10 expression at the site of disease [14–19]. The pathogenesis of RR may involve Th17 cells [20] although the specificity of this response remains to be determined [21]. RR is also characterized by a reduction in M2 macrophages along with a decrease in IFN-β expression and an increase in M1 macrophages in skin lesions [22, 23].

RRs involve a change in immune status towards *M*. *leprae* that leads to a reduction in the number of bacilli in disseminated lesions, such that investigators have sought to identify the dynamic changes in the systemic immune response. Studies have included the measurement of several cytokines in serum [24–27] as well as the detection of specific cytokine mRNAs associated with the onset of RR [28, 29]. Here, we used an unbiased approach to assess intra-individual gene expression changes associated with RR, by measuring the peripheral blood transcriptome and associated gene networks in longitudinal samples obtained from leprosy patients before the onset of RR, at the time of RR diagnosis and after treatment for RR resulting in resolution of the reactional state.

## Methods

### Patients and clinical specimens

Peripheral blood was collected from leprosy patients of various endemic regions: Bangladesh (International Centre for Diarrhoeal Disease Research Bangladesh, Dhaka), Brazil (National Reference Centre for Sanitary Dermatology and Leprosy, Uberlandia and Leprosy Laboratory), Ethiopia (ALERT hospital and Health Centre, Addis Ababa) and Nepal (Mycobacterial Research Laboratories, Kathmandu). The MB patients recruited for this study did not include pure LL (lepromatous) patients, but mostly the clinically unstable borderline patients, including BB (mid-borderline), BL (borderline lepromatous) as well as BL/LL. These patients with borderline leprosy are more likely to develop RR during MDT. BT (borderline tuberculoid) patients were designated paucibacillary (PB) (S1 Table).

For longitudinal studies, 3 samples from ten patients who developed RR during multidrug therapy (MDT), were included. Clinical monitoring for reactions was performed during monthly clinic visits. For longitudinal sample recruitment of reactional patients, newly diagnosed, untreated leprosy patients without clinical reactions were enrolled and blood was drawn before initiation of MDT (BR, previously referred to as t = 0, [29]). Patients who presented reactions within three months of the start of therapy were excluded from the cohort to avoid analyses of latent reactions. If patients presented with reactions after more than three months of MDT, blood was drawn at the time of diagnosis of RR, before initiation of therapy to suppress the reactional state. From all patients, blood was collected after completion of steroid therapy (AT, previously referred to as t = end, [29]) (S2 Table). Also, 16 (8 MB and 8 PB) leprosy patients without leprosy reactions at the time of recruitment and at the end of MDT were included as a control group (S1 Table).

### Ethics statement

All leprosy patients were recruited with approval from the Institutional Review Board and the Institutional Ethics Committee of all institutions listed above. All subjects provided a written informed consent and a parent or guardian of any child participant provided informed consent on the child’s behalf.

### Peripheral blood collection and RNA isolation

Peripheral blood (2.5ml) was collected in PAXgene tubes (BD Biosciences, Franklin Lakes, NJ) for 46 leprosy samples defined as multibacillary (MB, containing BB, BL and LL patients), paucibacillary (PB, containing BT patients), before reaction (BR), reversal reaction (RR) and after treatment for RR (AT). Total RNA was isolated from venipuncture using PAXgene blood collection tubes and stored at -80°C. RNA isolation was performed using the PAXgene Blood RNA kit (BD Biosciences) including on-column DNase digestion according to the manufacturers’ protocol [29]. The RNA amount from all samples was determined by a NanoDrop ND-1000 spectrophotometer (NanoDrop Technologies, Wilmington, DE) and samples yield average of 6.02 ± 1.5 μg, with an average OD260/280 ratio of 2.0 ± 0.04. The RNA quality and integrity were accessed by Agilent 2100 BioAnalyzer using the RNA 6000 Nano Chip kit. The average RIN (RNA integrity number) of the total RNA samples obtained from PAXgene tubes was 9.5 ± 0.08.

### RNA sequencing

Total RNA (100 ng per sample) from 46 samples was subjected to poly-A-selection to purify messenger RNA, then fragmented and converted into double-stranded cDNA. Library construction was then carried out using the TruSeq Sample Preparation Kit (Illumina) according to the manufacturer’s instructions. This included the ligation of sequencing adapters containing 7 nucleotide indexes for multiplexing. Libraries were quantified using PicoGreen (Invitrogen) and quality was assessed using the Agilent 2200 Tapestation. Library samples were pooled (6 per lane) at equimolar quantities (10uM each library) and sequenced on a HiSeq 2000 sequencer (Illumina) with 100bp single-end protocol [30]. All relevant data are available from the NCBI GEO repository database and are accessible through GEO series accession number GSE120913. https://www.ncbi.nlm.nih.gov/geo/query/acc.cgi?acc=GSE120913

### Bioinformatics analysis

Sequenced reads were demultiplexed and aligned to the human reference genome hg19 (UCSC) using TopHat (version 2.0.6) and Bowtie2 (version 2.0.2) as previously described [30]. The HTseq package was then used to assign uniquely mapped reads to exons and genes using the gene annotation file for build hg19 from Ensembl to generate raw count data. Once raw count data was generated, data normalization and differential expression analysis using a negative binomial model were performed in the R statistical programming environment using the DESeq (version 2.0) Bioconductor package. Significant differentially expressed genes were defined as fold change >1.2 between groups and P-value <0.05.

### Hierarchical clustering and principal components analysis (PCA)

PCA and hierarchical clustering were performed using ClustVis [31]. Only genes with average normalized counts of ≥ 2.0 were included and a cutoff of a coefficient of variance of ≥ 1.0 was used to perform both analyses.

### Weighted gene correlation network analysis (WGCNA)

Gene expression profiles were obtained and analyzed for modules of highly interconnected genes using weighted gene correlation network analysis (WGCNA), an unbiased approach that identifies the typical pattern of each cluster (the eigengene) and defines modules of highly interconnected genes based on pairwise correlations [32]. The analysis was performed on the filtered leprosy subtype gene expression profiles (“wgcna” package in R). Automatic network construction was carried out with a power of 14 and a minimum module size of 50. For each module, networks were constructed using the topological overlap matrix. The top 50 genes from each network were selected by filtering using kME (intramodular connectivity) and converted to gene names before displaying. Module correlation of RR temporal stages was calculated by computing the correlation of each module eigengene to a binary matrix of traits, which corresponded to individual stages [33]. Correlation and significance calculations, as well as heatmap display, were calculated using built-in functions from the “wgcna” R package. P values for overlap of modules with cell-type-specific signatures were calculated using the hypergeometric distribution and were corrected using a Bonferroni adjustment (n = 30).

### Functional and upstream regulator analyses

Functional, network and transcriptional regulator analysis were performed using the ClueGO plugin (Cytoscape) [34] and Canonical Pathway Analysis (IPA). The Canonical Pathways display the most significant pathways across the entire dataset. The significance values for the canonical pathways are calculated by B-H Multiple testing p-value. The significance indicates the probability of association of molecules from your dataset with the canonical pathway by random chance alone. ClueGO integrates Gene Ontology (GO) terms as well as KEGG pathways and creates a functionally organized GO/pathway term network. The significance of each term was calculated with a right-sided hypergeometric test with B-H correction of p-values. Significantly overrepresented terms were defined as having a p-value less than 0.05, a minimum of 4 genes per term, and at least 6% of the genes from the dataset associated with the term. Functionally similar GO terms were grouped into simplified representative terms.

IPA network analysis was performed to investigate the top gene networks related to a gene set. Briefly, networks are collections of interconnected molecules assembled by a network algorithm; each connection represents known relationships between the molecules, found in the Ingenuity Knowledge Base. IPA Upstream Regulator Analysis was used to identify upstream regulators and predict whether they are activated or inhibited, given the observed gene expression changes in our experimental dataset. The analysis examines the known targets of each upstream regulator in a dataset, compares the targets’ actual direction of change to expectations derived from the literature, then generates a prediction for each upstream regulator. IPA uses a z-score algorithm to make predictions. The z-score algorithm is designed to reduce the chance that random data will generate significant predictions. The interactive visualization platform Gephi was used for visualization of integrative analysis of functional networks and upstream regulator analysis.

### Interferon signature analysis by SaVanT

The interferon signature was calculated using signatures containing 50 genes for each stimulus, signature scores were calculated for each stimulus based on the mean score of the log-transformed, mean-centered values. The scores for each signature were then clustered based on average Euclidian distance. All p values and scores were calculated using a gene signature-based analysis in SaVanT (Signature Visualization Tool) [35].

### Proportional median signature

In order to identify genes that were highly expressed in one subtype relative to all others, we calculated proportional median values for all filtered probe sets in every subtype. Briefly, the proportional median is a measure for comparing three or more conditions, and it is calculated for gene in each disease by dividing the median expression of that gene in that disease by the median expression of that same gene across the disease subtypes. Thus, ranking genes by their proportional median measures the relative expression in one subtype compared with all others [36, 37]. Proportional median values were calculated for each leprosy state (MB, BR, RR, and PB) and the top 500 genes from each PM list were used for further analysis.

### Specific interferon regulated genes

Supervised analyses were performed to identify Type I and Type II IFN regulated genes as described previously [23]. Differentially expressed genes between RR and BR/AT leprosy groups were identified with a pairwise comparison using the criteria of a fold change >1.2 and p ≤ 0.05. In addition, we used the top 500 PM genes for each leprosy state for the analysis of the specific interferon regulated genes. A list of genes specifically induced by only IFN-β or IFN-γ was derived from the gene expression profile data of IFN-treated MDMs (GSE82227 and GSE125352). Briefly, MDMs were treated with IFN-γ and IFN-β at different time points, the max fold change was calculated between IFNs vs. media for all time points. To identify the downstream specific genes for IFN-γ and IFN-β we used a cutoff of 2-fold and padj of 0.05 for IFNs vs. media. For genes that were upregulated by both IFNs, only genes with a ≥5 fold change between IFN-γ and IFN-β and vice versa were used to generate the specific lists. We identified a total of 245 IFN-β specific genes and 50 IFN-γ specific genes, these IFN downstream gene targets were then integrated with the leprosy whole blood RNAseq data to determine the differential expression of IFN-regulated genes in different disease forms. Enrichment analysis of the overlap in IFN target genes between the different leprosy datasets was performed using the hypergeometric distribution to control for differences in the overall number of differentially expressed genes. The hypergeometric distribution (hypergeometric test) is equivalent to the one-tailed version of Fisher's exact test. The IFN summation score was calculated using a gene voting approach based on the sum of the signed log ratio of the normalized counts of the IFN-β specific and IFN-γ specific induced (positive in the summation) genes in each blood sample as previously described [23, 38].

### GBP expression values

We measured the expression of the GBP (GBP1 to GBP6) mRNAs using previously generated and publicly available data: in blood (normalized counts; GSE120913), in skin (arbitrary units; GSE17763 [23]) of leprosy patients, as well as, in MDM treated with IFN-γ, IFN-β and TLR2/1 ligand (normalized counts, GSE82227 and GSE125352).

### PCR

Total RNA was isolated from whole peripheral blood of 46 leprosy patients (S1 Table) as described in previously at ‘Peripheral blood collection and RNA isolation’ section. The cDNA was prepared as previously described [23, 38] and gene expression levels of human GBP1, GBP2, GBP3, GBP4, GBP5, GBP6 and, 36B4 were measured by qPCR and calculated by the 2^-(ΔΔCt)^ method. GBP primers were designed using BLAST and to avoid genomic DNA recognition the reverse primers were designed in an exon junction. Primer information and sequence are listed in S5 Table:

### Statistical analysis

All differential expression analysis was performed in the R statistical programming environment using the DESeq (version 1.5) as explained in the Bioinformatic analysis section. Results are reported as pooled data from an entire series of experiments and described as mean ± the SEM unless otherwise indicated. The GraphPad Prism 7 software was used for testing of parametric distributions, statistical and correlation analysis. We performed Kolmogov Smirnov normality test and equality of variances test (Bartlett's test) on values to verify whether the data were parametrically distributed. For data with one grouping found to have a parametric distribution, statistical analysis was performed by one-way analysis of variance ANOVA followed by the Turkey multiple comparison posttest. For data with two grouping variables, one defined by columns and the other defined by rows we used two-way analysis of variance ANOVA followed by the Turkey multiple comparison posttest. Pearson correlation was used for correlation analysis between RNAseq and qPCR data. The two-tailed student t test was used to evaluate the correlation significance. Individual details of statistical analyses are explained in the figure legends.

## Results

### Gene expression of whole blood of leprosy patients on reversal reaction

We performed a longitudinal transcriptome analysis of blood from patients with RR to identify the mechanisms that contribute to cell-mediated immunity in leprosy (S1 Fig). RNA was isolated from the whole blood of 10 leprosy patients obtained at the time of the initial diagnosis of leprosy (before reaction; BR), at the time of the diagnosis of reversal reaction (RR) and after the completion of treatment of RR with prednisone (after treatment; AT), these time points were named RR temporal stages (S2 Table; S2A Fig). RNA sequencing of these 30 samples was simultaneously performed. The background expression was filtered yielding a dataset of 12,649 genes expressed genes.

Unsupervised testing, including principal component analysis (PCA) and hierarchical clustering, was performed on a set of genes that was selected using the coefficient of variance of the normalized RPKMs of the 10 patients in the different RR temporal stages (BR, RR and AT). PCA revealed a heterogeneous distribution of all samples across the first two principal components as seen by the overlap of the ellipsoids representing each group (S2B Fig). Hierarchical clustering showed the distribution of samples is largely determined by the geographic region and by an individual patient. We observed the patients from two main geographic regions, Bangladesh (n = 4) and Brazil (n = 3), are organized in different branches in the hierarchical tree (S2C Fig). For six of the ten samples derived from the same patients all three RR temporal stages are clustered together (S2C Fig). For three other patients, two of the three time points clustered together. These data suggest that genetic differences between individuals rather than temporal changes for each individual accounted for the overall differentiation of the transcriptomes.

### Functional analysis of differentiated expressed genes in the blood of RR patients

**Fig 1:**
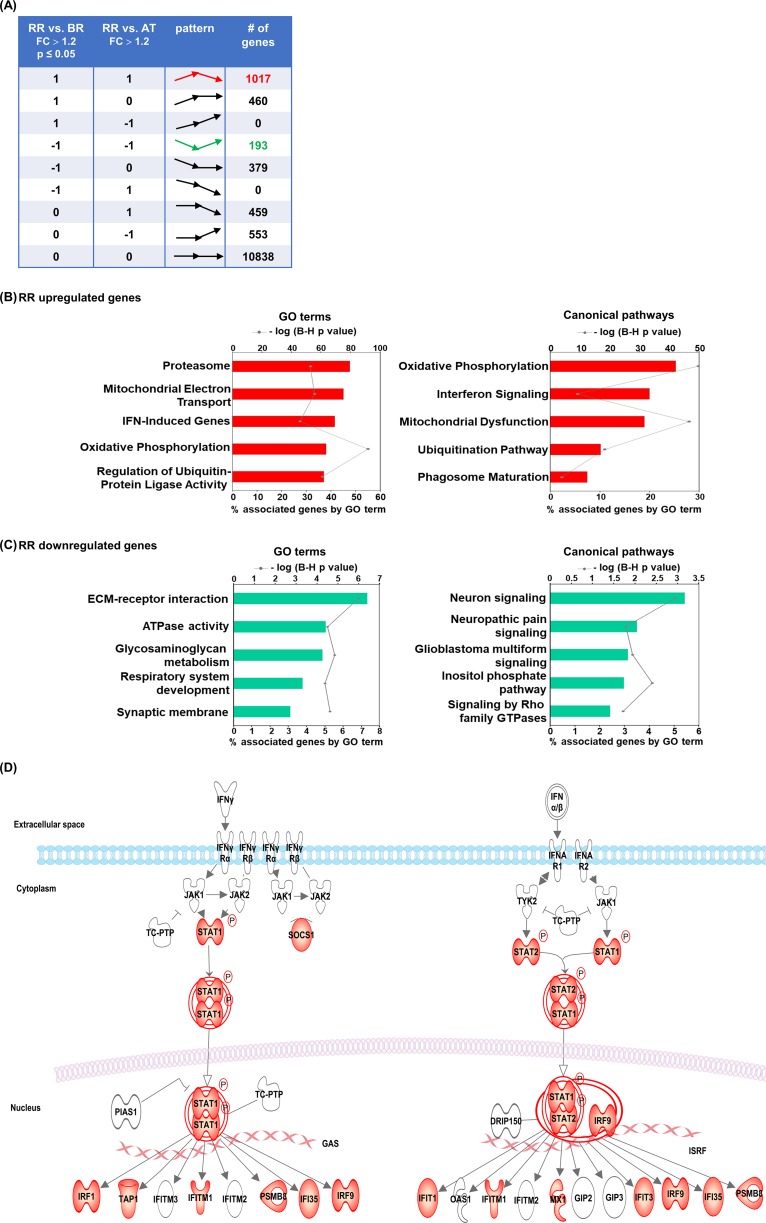
Functional analysis of differentially regulated genes. (A) For differential gene expression between RR, BR and AT whole blood samples we calculated fold change analysis and performed a paired statistical test using DEseq2. A cut of fold change ≥ 1.2 and p ≤ 0.05 was applied for RR vs. BR and fold change ≥ 1.2 was applied for RR vs. AT. In examining the RR temporal stages: BR (before reaction); RR (reversal reaction), AT (after treatment), we identified 1017 genes upregulated in RR vs BR and AT and 193 genes downregulated in RR vs. BR and AT. Top 5 GO terms and canonical pathways identified by ClueGO and IPA respectively of significantly upregulated genes (B) and downregulated genes (C) for RR whole blood samples. Graphs show the number of associated genes and -log p-value for each GO terms. Padj was calculated with B-H multiple testing for the association of the functional term with the gene-expression data. (D) Upregulated RR genes in the IPA interferon signaling canonical pathway are presented in red.

Because the genetic variation among patients was a strong driver of the sample clustering, we chose to make pair-wise comparisons for each patient to determine the changes in gene expression that were common among the diverse genetic background of the patients studied. This was performed by comparing the gene expression for each of the RR temporal stages; at the time of leprosy diagnosis (BR), at the time of the first RR (RR) and after prednisone treatment (AT). To more precisely identify the differentially expressed genes associated with the development of RR, we calculated the fold change of individual genes comparing the gene expression value at the time of the initial diagnosis of RR as compared with before reaction (BR) and after treatment (AT). In addition, a paired statistical test was used for p-value calculation. Because the genetic diversity of the samples, we used a less stringent cutoff of fold change >1.2 and a p-value ≤ 0.05. We identified an ‘RR signature’ comprised of 1017 genes that were upregulated during RR in comparison with BR and AT. Only 193 genes were downregulated during RR in comparison with BR and AT (Fig 1A, S3 and S4 Tables). Clustering and PCA analyses (S2 Fig) showed that samples from the same patients were more similar to each other as compared to the differences between the different BR, RR and AT groups, such that it was not possible to use a padj, similar to another study of peripheral blood transcriptomes in leprosy [39].

Functional analysis of the RR upregulated genes identified multiple significantly enriched GO terms and canonical pathways. The top five GO terms were, ‘proteasome’, ‘mitochondrial electron transport’, ‘IFN induced genes’, ‘oxidative phosphorylation’ and ‘regulation of ubiquitin-protein ligase’ (Fig 1B). Canonical pathway analysis showed similar results to GO terms, including ‘oxidative phosphorylation’, ‘interferon signaling’, ‘mitochondrial dysfunction’, ‘ubiquitin pathway’ and ‘phagosome maturation’ (Fig 1B). Similar to the GO term findings, canonical pathway analysis revealed that ‘IFN signaling’ was one of the top pathways, and most of the genes included in the pathway are upregulated in the RR blood samples (Fig 1B). Because there were fewer downregulated genes, the functional analysis identified relatively few GO terms and canonical pathways; however, both analyses showed metabolism and neural terms and pathways (Fig 1C). The ‘IFN signaling’ canonical pathway is enriched in the RR signature (Fig 1D).

### Interferon signaling is upregulated during reversal reaction

**Fig 2:**
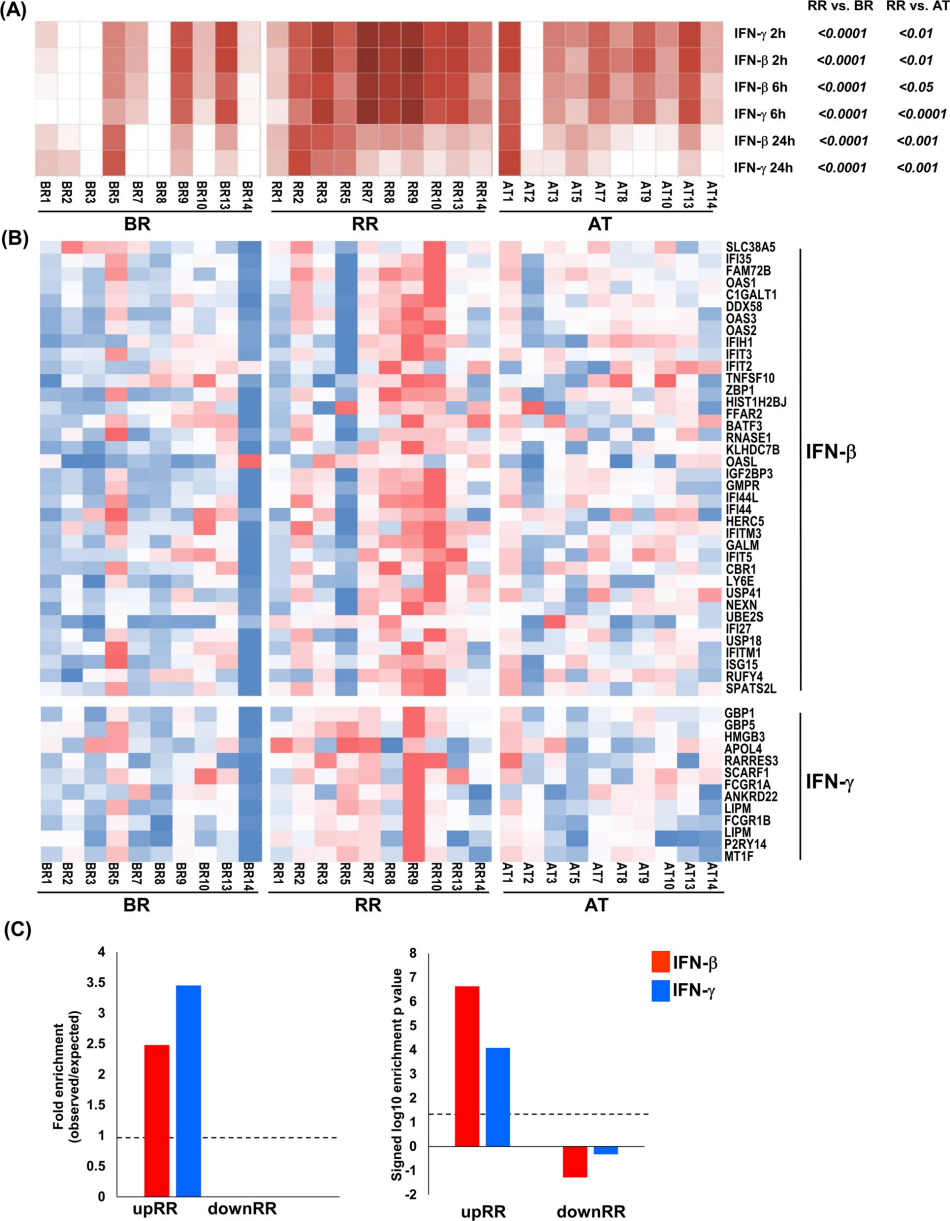
IFN-β and IFN-γ signature on RR samples. (A) IFN-β and IFN-γ gene expression signature by SaVant. For each time point of the IFN-β and IFN-γ MDM gene expression data, signature enrichment scores were calculated using average gene expression for each RR temporal stages and normalized to Z scores. Columns correspond to RR temporal stages and rows correspond to IFN signature; each individual square corresponds to the enrichment for one IFN signature in a specific RR sample for each subtype. P-value was calculated by one-tailed ANOVA, followed by Tukey's multiple comparisons test. (B) Heatmap showing the normalized counts for the 38 and 13 overlapped genes between RR upregulated genes and IFN-β and IFN-γ MDM specific genes, respectively. (C) Enrichment analysis of overlap between IFN-β and IFN-γ- specific upregulated genes identified in human MDMs and RR and BR/AT specific leprosy whole blood transcripts (fold change ≥ 1.2 and P ≤ 0.05). Dotted lines indicate either the expected fold enrichment of one (left) or the hypergeometric enrichment p-value of 0.05 (log P = 1.3, right). Hypergeometric analyses were performed to determine fold enrichment (observed/expected) and signed log enrichment p- value (negative for de-enriched). The Bonferroni multiple hypothesis test correction was applied for each group. RR temporal stages: BR = before reaction, RR = during reversal reaction and AT = after treatment. N = 10 for each RR subtype.

Given that the functional analysis identified IFN-induced genes and ‘IFN signaling’ as significant signatures detected in the blood of patients during RR, we further evaluated the IFN-β and IFN-γ pathways using a gene signature-based analysis in SaVanT (Signature Visualization Tool) [35]. SaVant contains the RNA-seq signatures from IFN-β and IFN-γ-stimulated human monocyte-derived macrophages (MDMs) [30] as a prototypic myeloid cell. We identified the IFN-β and IFN-γ signatures present in each sample and found that both the IFN-β and IFN-γ signatures increased significantly in the blood of patients during RR in comparison to BR and AT. Both the IFN-β and IFN-γ signatures at 6h and 24h were strongly correlated to RR (Fig 2A).

The Savant gene signatures for IFN-β and IFN-γ overlap given that both IFNs signal via STAT1; however, IFN-β signals via a STAT1/2 heterodimer and IFN-γ signals via a STAT1 homodimer. We therefore integrated the IFN-specific gene signatures derived from stimulated monocyte-derived macrophages with the RR transcriptomes. Of the total 1017 upregulated genes in the RR signature, 38 genes overlapped with the 245 genes in the IFN-β signature and 13 genes overlapped with the 50 genes in the IFN-γ signature (Fig 2B). No IFN specific genes were detected in the 193 downregulated genes associated with RR. Both the IFN-β-specific genes and the IFN-γ-specific genes were significantly enriched in the RR signature (p = 2.3e-08, and p = 8.3e-05, respectively, Fig 2C).

**Fig 3:**
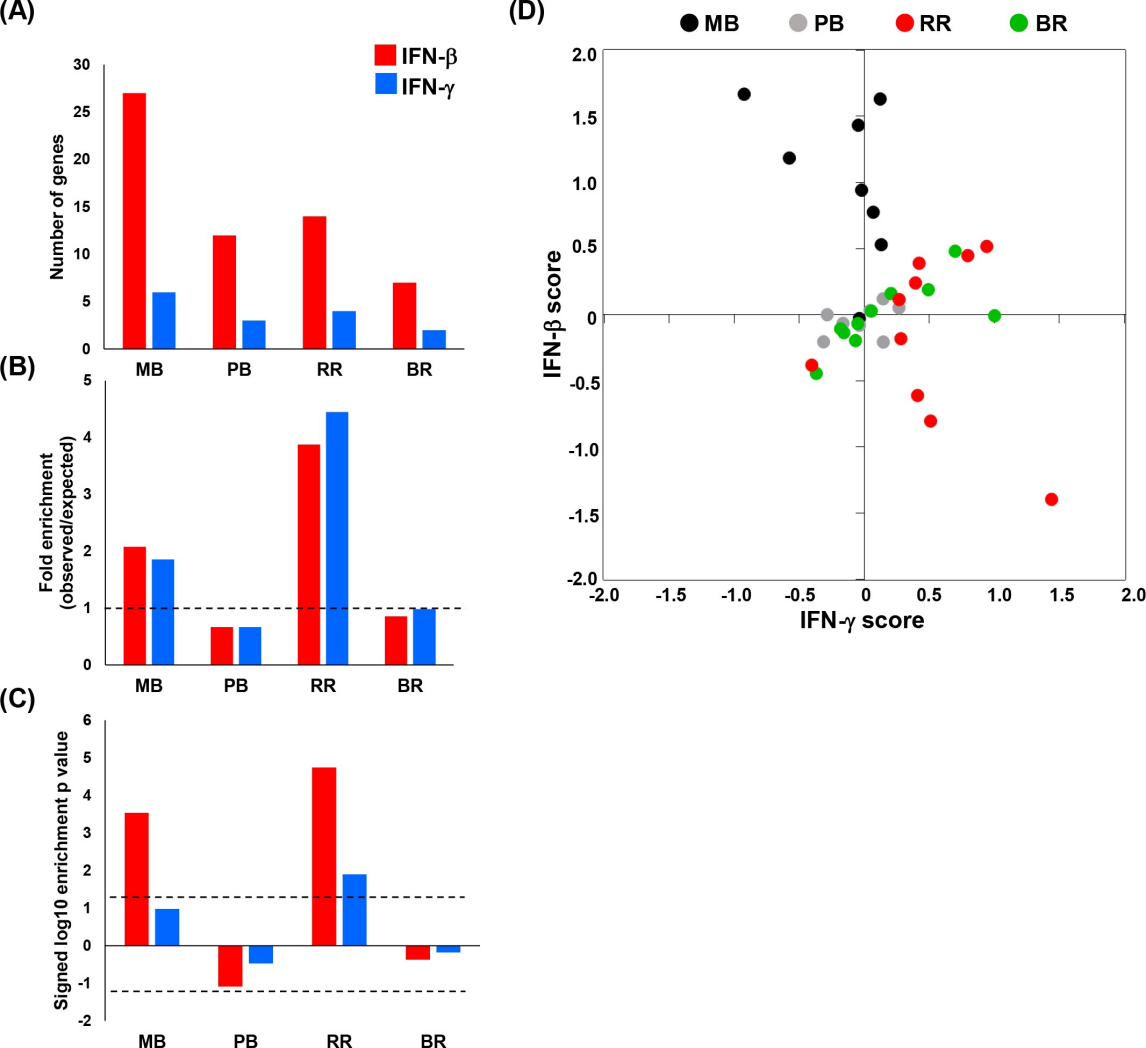
IFN-β and IFN-γ specific downstream gene signature for all leprosy subtypes. (A-C) Enrichment analysis of overlap between IFN-β and IFNγ–specific upregulated genes identified in human MDMs and MB, BR, RR and PB leprosy whole blood transcripts (PM = top 500 genes). The top graph shows the number of overlapped genes between each leprosy clinical form and IFN-β and IFN-γ specific genes (A). Dotted lines indicate either the expected fold enrichment of one (middle—B) or the hypergeometric enrichment p-value of 0.05 (log P = 1.3, bottom—C). Hypergeometric analyses were performed to determine fold enrichment (observed/expected) and signed log enrichment p-value (negative for de-enriched). The Bonferroni multiple hypothesis test correction was applied for each group. (D) IFN-β and IFN-γ specific gene voting summation scores were calculated for an individual patient blood sample in leprosy states MB (n = 8), BR (n = 10), RR (n = 10) and PB (n = 8). MB = multibacillary, BR = before reaction, RR = reversal reaction and PB = paucibacillary.

To define the pattern of the IFN signatures in whole blood of leprosy patients in more detail, we compared the IFN-β and IFN-γ- gene signatures in the different clinical forms of leprosy. In addition to the patients with the RR temporal stages (BR, RR and AT) we performed RNA sequencing on 16 whole blood control samples from multibacillary (MB, n = 8) and paucibacillary (PB, n = 8) patients (S1 Table). The MB and PB patients did not develop a leprosy reaction during the follow-up of this study (2 years). To compare the IFN-β and IFN-γ-specific signatures between the four different disease subtypes (BR, RR, MB and PB) we calculated the proportional median (PM) from the genes normalized counts for each group as described in the methods [36, 37]. We used the top 500 PM genes for each group. Integration of the different leprosy blood gene expression profiles with the IFN-induced gene signatures (Fig 3A) revealed the significant enrichment of IFN-β genes in the MB gene expression profile (fold enrichment = 2; p = 2.9e-04), but not IFN-γ genes (Fig 3B and 3C). For the RR blood samples, the enrichment for both IFN-specific signatures was significantly greater than expected; the IFN-β specific signature was enriched by 3.9-fold (p = 1.8e-05) and the IFN-γ specific gene signature was enriched by 4.5-fold (p = 13e-02) (Fig 3B and 3C). No significant enrichment for the IFN specific signatures was observed in the PB and BR whole blood samples (Fig 3C).

Finally, to evaluate the IFN status of each individual whole blood sample, we calculated an overall score of the IFN-β and IFN-γ regulated genes using a gene voting approach [23, 38]. Across the leprosy subtypes, we observed differences in the IFN-β and IFN-γ scores. Seven of the eight MB patients had a positive IFN-β score, with five of eight distinguished as having a more positive score than all other patient samples (Fig 3D). Nine of ten RR patients showed positive scores for the IFN-γ-induced gene signature; five of which were also positive for the IFN-β-induced gene signature (Fig 3D). Three of ten BR patients had positive IFN-γ scores within the range of the five highest RR patients and greater than any of the PB and MB patients. Otherwise, the IFN-signatures of the BR and PB patients were located close to the origin (Fig 3D).

### Identification of gene modules and deconvolution analysis for reversal reaction stages

**Fig 4:**
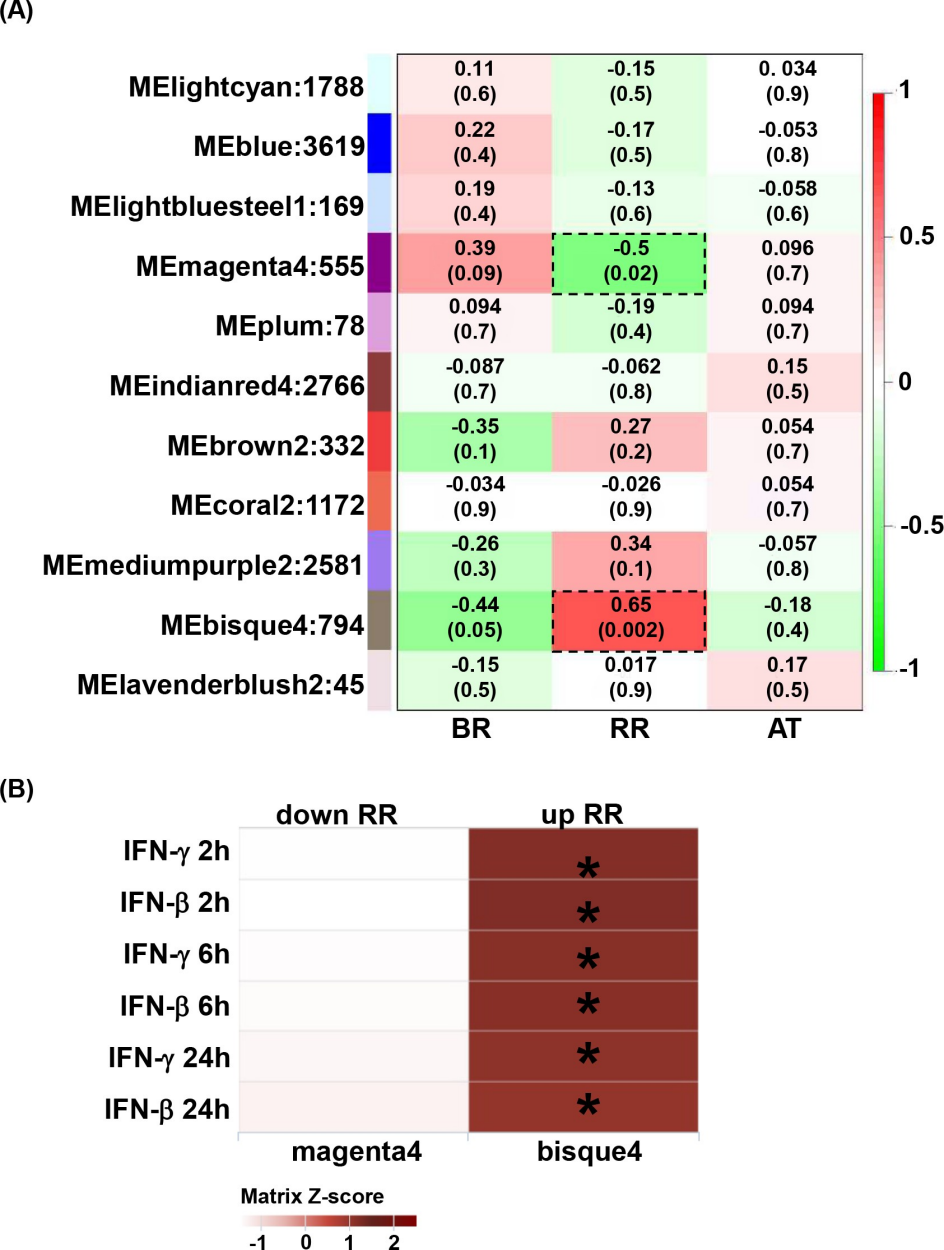
Gene expression in whole blood from RR temporal stages. (A) Identification of RR whole blood subtype gene modules. WGCNA eigengene modules correlated to at least one RR temporal subtype (p ≤ 0.05). Red indicates a positive correlation, and green indicates an inverse correlation. Module eigengenes, as well as the corresponding number of genes in each module, are labeled on the y axis, and RR temporal stages are labeled on the x axis. BR (before reaction), RR (reaction) and AT (after reaction treatment). (B) Integration of WGCNA gene modules with cell-type-specific gene signatures. For the two significant modules derived from WGCNA, enrichment for MDM IFN-β and IFN-γ specific downstream genes (2h, 6h and 24h) were calculated and displayed in a heatmap of Z scores. Hypergeometric analyses were performed to determine enrichment p-value. * p<0.001.

The overlap of IFN induced gene signatures with the peripheral blood transcriptomes from leprosy patients suggested that specific gene networks are triggered during RRs. To identify modules of interconnected genes in the peripheral blood transcriptomes of leprosy patients, we applied an unsupervised approach, weighted gene correlation analysis (WGCNA). WGCNA is an unbiased approach that identifies the typical pattern of each cluster (the eigengene) and defines modules of highly interconnected genes based on pairwise correlations [32]. WGCNA identified 11 modules using all the transcriptome data from the blood of leprosy patients in the different RR temporal stages of RR (BR, RR and AT). Two module eigengenes (ME) were significantly associated with RR, but none of the modules were significantly correlated with BR and AT. The *bisque4* module was positively correlated with RR (correlation = 0.65, P = 0.002) and the *magenta4* module was negatively correlated with RR (correlation = -0.5, P = 0.02) (Fig 4A).

To further explore whether the WCGNA gene modules that correlated with RR were enriched for an IFN-downstream network, we integrated the *bisque4* and *magenta4* modules with the signatures derived from IFN-treated MDMs using SaVant. The module *bisque4* that positively correlated with RR, significantly correlated with both the IFN-γ and IFN-β signatures (hypergeometric enrichment p-value ≤ 0.001 for all the IFN treatment time points, (Fig 4B). The *magenta4* module that negatively correlated with RR did not correlate with any IFN gene signature.

### Interferon signaling drives the immune response in the blood of RR patients

The RR gene expression signature contains genes that were differentially expressed compared to BR and AT but does not identify gene networks. By contrast, WGCNA identifies gene networks, but not all the genes in a given module are differentially expressed in a particular disease type. Therefore, we integrated the differential gene expression analysis with the correlated WGCNA modules to identify genes that were part of a gene network and differentially expressed in RR. We identified 434 common genes that were upregulated in RR signature and the *bisque4* module (hypergeometric analysis identified a fold enrichment of 15.38 and P = 2.0e-376 (S3 Fig). The *bisque4* module and the genes downregulated in RR vs. BR/AT did not share any genes. For the *magenta4* module, 25 genes were found in common with those downregulated in RR (fold enrichment of 5.36 and P = 1.6e-10). The *magenta4* module did not contain any genes upregulated in RR. Because there were only 25 common genes between RR downregulated genes and *magenta4*, it was not possible to use ClueGO or IPA to perform functional analyses.

**Fig 5:**
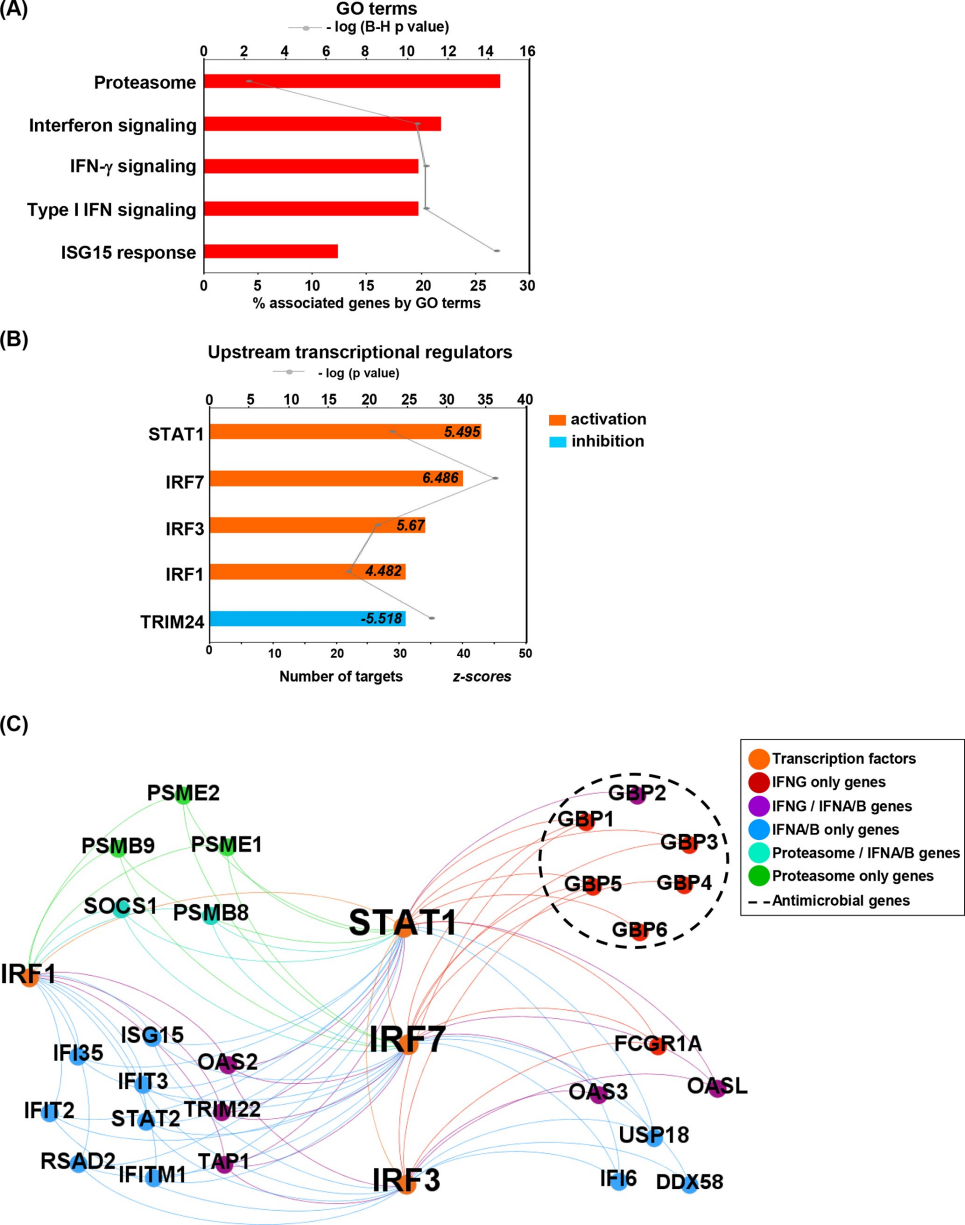
Functional analysis (434 common genes between bisque4 and upregulated genes). (A) Top 5 functional GO terms for the 434 common genes between the RR positively correlated module and RR upregulated genes. Graphs show the number of associated genes, -log p-value and 5 hits for each GO term. Padj was calculated with B-H multiple testing for the association of the functional term with the gene-expression data. (B) Top 5 upstream transcriptional factors for the 434 common genes. The upstream analysis was performed by IPA upstream regulator analysis. Graphs show the number of target genes, -log p-value and z-score for each transcriptional factor. Orange and positive z-score for activation and blue and negative z-score for inhibition. (C) Integrated network of gene expression, upstream regulators and functional analysis terms. Gephi was used to create a functional annotation network, showing connections among significant GO terms, IPA upstream regulator analysis and 434 common genes between bisque4 module and upregulated significantly expressed genes. Genes are colored by term a, transcription factors in orange and connections are represented by dotted lines.

We performed ClueGO functional analysis of the 434 common genes between the *bisque4* module and the genes upregulated in RR. The top five classified GO terms were ‘proteasome’ (28% of the associated genes), including *PSMA1*, *PSMA5* and *PSMB2*; ‘interferon signaling’ (23%), including *GBP2*, *IFI27* and *IFI35*; ‘IFN-γ signaling’ (20%), including, *GBP1*, *GBP2* and *GBP5*; ‘type I IFN signaling’ (19%), including *ISG15*, *OAS2* and *OAS3*; and ‘ISG15 response’ (13%); including *DDX58*, *FCGR1* and *GBP1* (Fig 5A). All of these pathways have been implicated in the immune response to infection by intracellular bacteria. Additional analysis of the “IPA upstream regulators” for the 434 common genes was performed. Briefly, we utilized two scores that address two independent aspects of the inference problem: an ‘enrichment’ score [Fisher’s exact test (FET) P-value] that measures the overlap of observed and predicted regulated gene sets, and a z-score assessing the match of the observed and predicted up/down regulation patterns. A positive z-score (≥ 2) indicates activation and a negative z-score (≤ -2) indicates inhibition. IFNG (z-score = 7.29, P = 3.91e-26) and IFNA (z-score = 6.32, P = 9.48e-31) as the top two cytokines of the upstream regulators. IRF7 (z-score = 6.486), IRF3 (5.67), STAT1 (5.495), IRF1 (4.482) and TRIM24 (-5.518) were identified as the top 5 transcriptional factors (Fig 5B).

To integrate the functional analyses on the 434 common genes between RR upregulated genes and the *bisque4* module, we overlapped GO terms and transcriptional targets and common genes between both analyses. We found that IRF7 (26/30 genes), STAT 1 (23/30 genes) and IRF1 (16/30 genes) upregulated genes related to all functions. We also identified proteasome (*PSMB* genes and *SOCS1*), IFNG signaling (*GBPs*, *OASs*, *FCGR1A*, *SOCS1* and *TRIM22*), interferon and IFNA/B signaling (*OASs*, *IFIs*, *GBP2*, *ISG15*, *STAT2*, *PSMB8*, *TAP1*, *USP18* and *RSAD2*). IRF3 (16 genes) regulated only IFNG signaling and IFNA/B signaling (Fig 5C). In addition to the analysis of GO terms and upstream regulators, we identified gene networks for the 434 RR genes using IPA. An antimicrobial network related to the RR blood gene signature was identified as one of the top networks. This network connects 38 genes with direct and/or indirect antimicrobial activity and 31 of them are present in the 434 RR genes, the two major groups identified in the antimicrobial network are “interferon inducible genes” and “guanylate binding proteins” (S4A Fig). This network also connected genes related to the IFN signaling pathways, IRF and STAT1 activation (*GBPs*, *DDX58*, *OAS2*, *ISG15*, *OASL*, *IFIs* and *IFITs*). To refine the antimicrobial network, we overlapped the 434 common genes in the RR transcriptome and the *bisque4* module with our curated antimicrobial gene list [30]. Only the guanylate binding protein (*GBP1*, *GBP2*, *GBP3*, *GBP4*, *GBP5* and *GBP6*) genes were identified as antimicrobial genes (S4B Fig, Fig 5C). Together the functional analysis, the upstream regulator analysis and the network analysis suggested a link between IFN signaling and GBP expression in RRs.

### GBPs are upregulated by IFN signaling during RR

**Fig 6:**
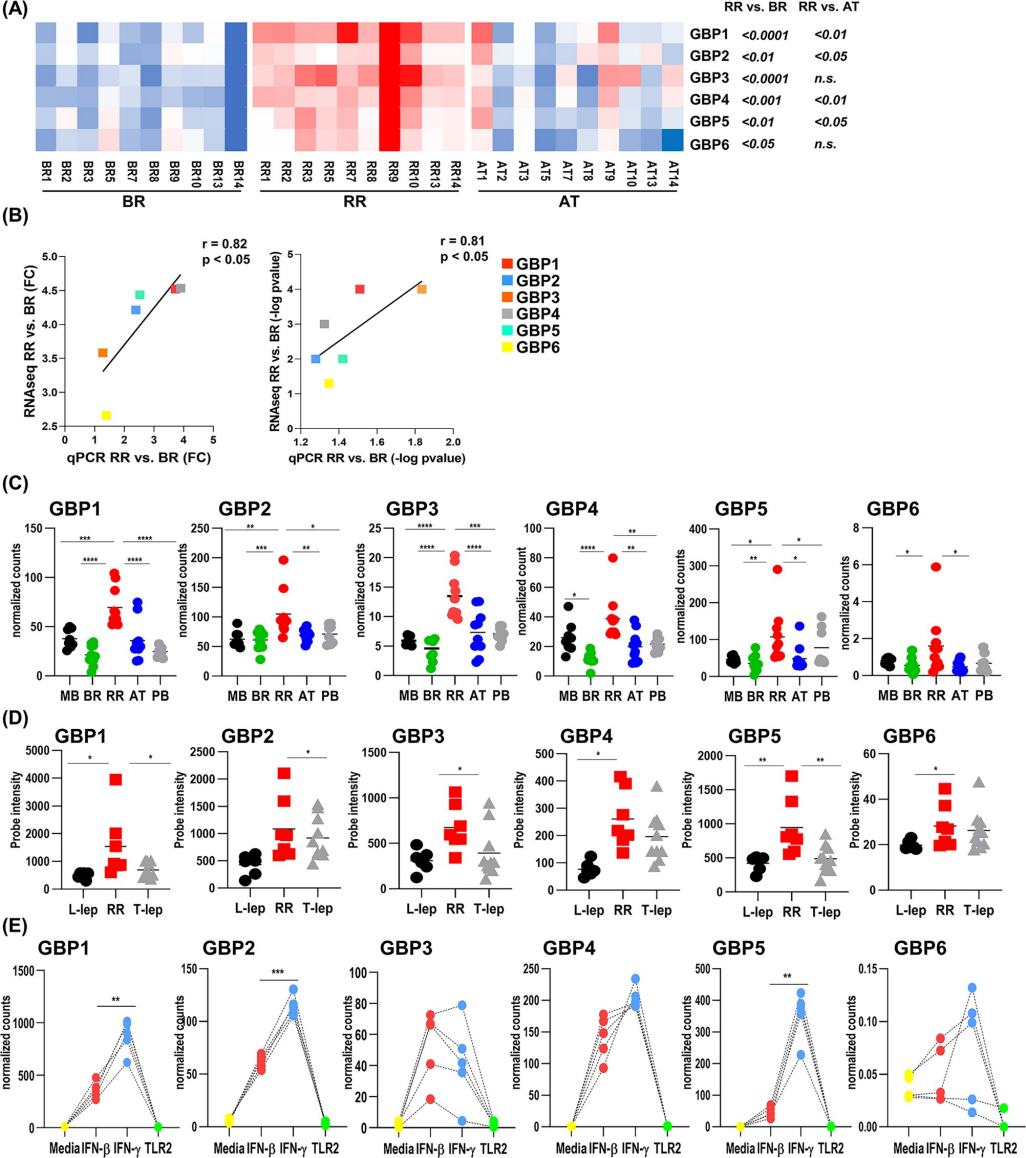
GBPs are upregulated in the peripheral blood of leprosy patients during RR. (A) Heatmap of GBPs 1 to 6 normalized counts from all 10 RR temporal stages (BR; RR and AT). Significance was determined by paired one-tailed ANOVA and post-hoc (Tukey multiple comparison test). (B) GBPs 1 to 6 correlation of fold change (FC) and p-value (-log p-value) between RNAseq and qPCR data. Squares represent the mean FC (RNAseq and qPCR) of the 10 RR pairs (RR vs. BR). Pearson correlation was used to calculate r values and the two-tailed p-value was calculated for correlation significance. (C) Distribution of GBPs 1 to 6 normalized counts in the whole blood for all groups of leprosy patients. The graph shows the mean per clinical type. MB = 8, BR = 10, RR = 10, AT = 10 and PB = 8 (D) Distribution of GBP 1 to 6 mRNA by microarray analysis of skin lesions shown in arbitrary units (AU). The graph shows the mean per clinical type. L-lep/MB = 6; RR = 7 and T-lep/MB = 10. Significance was determined by one-tailed ANOVA using GraphPad Prism software and post-hoc (Tukey multiple comparison test). (E) Distribution of GBPs 1 to 6 mRNA in the RNAseq analysis of human MDMs stimulated for 24h, media, yellow; IFN-β, red; IFN-γ, blue and TLR2/1 ligand, green. The graph normalized counts for all 5 individuals per stimuli for GBPs 1 to 6. Significance was determined by paired one-tailed ANOVA using GraphPad Prism software and post-hoc (Tukey multiple comparison test). * P <0.05, **P < 0.01, ***P < 0.001, ****P < 0.0001. MB = multibacillary, BR = before reaction, RR = reversal reaction, AT = after treatment, PB = paucibacillary, L-lep = lepromatous leprosy and T-lep = tuberculoid leprosy.

To visualize GBP expression as a function of the kinetics of RR, we constructed a heatmap for GBP (*GBP1*, *GBP2*, *GBP3*, *GBP4*, *GBP5* and *GBP6*) expression using normalized counts for the 10 different RR patients with the RR temporal stages (n = 30; 10 BR, 10 RR and 10 AT). All GBPs are upregulated during the RR in comparison to BR and downregulated AT for all ten donors except for patient 1, in which the levels of GBPs were maintained from RR to AT (Fig 6A).

To validate the RNAseq data we performed qPCR for all six *GBP* mRNAs for the RR temporal stages samples previously used in Fig 6A. We verified that *GBP1*, *GBP2*, *GBP3*, *GBP4*, and *GBP5* were upregulated during RR in comparison to BR and AT; *GBP6* was only upregulated during RR in comparison to BR but did not decrease in AT (S5A Fig). A significant correlation in GBP expression was verified between the RNAseq normalized counts and qPCR arbitrary units across the RR temporal stages for all RR patients (S5B Fig). In addition, significant correlations were observed when the RNAseq and qPCR data were compared according to fold change and p-value for all six GBPs (Fig 6B). To verify that the differential expression of all GBPs was significantly upregulated during RRs, we first compared the expression of the GBPs (*GBP1*, *GBP2*, *GBP3*, *GBP4*, *GBP5* and *GBP6*) in the different RR temporal stages (RR, BR and AT) and control (PM and MB) sets). *GBP1*, *GBP2*, *GBP3* and *GBP5* were significantly higher in RR in comparison to MB, BR, AT and PB with *GBP4* significantly higher in RR compared to BR, RR and PB, *GBP6* significantly higher in RR compared to BR and AT (Fig 6C).

To confirm the RNAseq results we measured GBPs gene expression levels in the same patient samples by qPCR (Fig 6B). *GBP1*, *GBP2* and *GBP5* were highly expressed in RR patients compared to MB, BR, AT and PB as previously detected by RNAseq (S5C Fig). A significant correlation in GBP expression was also verified between the RNAseq normalized counts and qPCR arbitrary units for all groups (S5D Fig). GBP mRNA expression was analyzed in the microarray skin lesion data across the leprosy spectrum (L-lep/MB, T-lep/PB and RR). All GBPs were upregulated in RR skin lesions in comparison to L-lep/MB skin samples and *GBP1* and *GBP5* were upregulated in RR in comparison to T-lep/PB (Fig 6D), consistent with the presence of an IFN-γ downstream gene signature in the paucibacillary RR and T-lep/PB skin lesions and an IFN-β downstream signature in the multibacillary L-lep/MB lesions [23].

Finally, in order to examine the ability of the IFNs to induce the GBPs, we assessed GBP gene expression in monocyte-derived macrophages (MDMs) following treatment with IFN-β, IFN-γ and TLR2/1 ligand (TLR2/1L) for 24h as measured by RNA sequencing and deposited in DermDB [36]. We verified that IFN-β and IFN-γ but not TLR2/1L induced *GBP1*, *GBP2*, *GBP3*, *GBP4* and *GBP5* expression in MDMs (Fig 6E).

**Fig 7:**
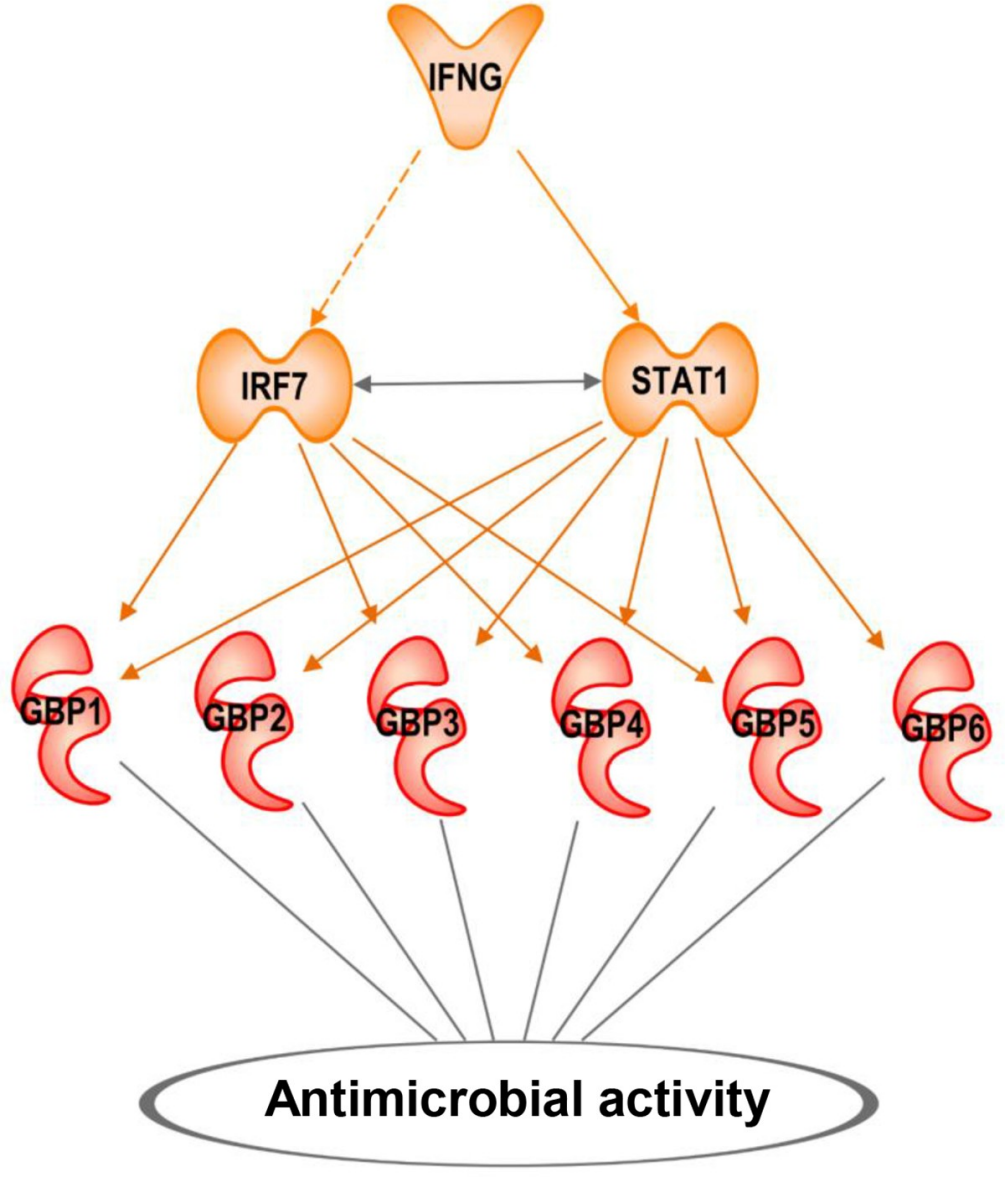
IFN-γ is the major upstream regulator for GBP expression in the RR peripheral blood. IPA pathway and path designer were used to create a functional IFNG downstream causal network. Upstream regulators with predicted activation are in orange and RR downstream upregulated GBPs are in red. Bold lines mean direct activation and dotted lines mean indirect activation.

The induction of *GBP1*, *GBP2* and *GBP5*, but not *GBP3* and *GBP4*, was significantly greater by IFN-γ than IFN-β. *GBP6* was not induced in MDMs. IPA analysis identified a pathway by which IFN-γ via IRF7 and STAT1 activate GBPs to mediate an antimicrobial response (Fig 7).

## Discussion

Reversal reactions in leprosy involve the rapid onset of cell-mediated immunity against *M*. *leprae* resulting in the reduction of the number of bacilli in lesions. The study of patients with RR represents a window into the dynamics of host defense against the disease-causing pathogen. Although the kinetics of the immune changes have been systematically studied in patient’s skin lesions [24–29], the breadth of immune changes in the peripheral blood reflecting the systemic response associated with the disease is not known. Here we identified a ‘RR signature’ of 1017 genes that were upregulated in the peripheral blood transcriptome at the onset of RR. This signature contained interconnected gene networks involving IFN-γ and IFN-β specific downstream genes. We detected a gene network correlated with RR, the *bisque4* module, enriched for genes in the RR signature that participate in antimicrobial responses including the IFN-γ induced genes *GBP1*, *GBP2* and *GBP5*, part of the guanylate binding protein family of antimicrobial genes. Therefore, RRs represent a systemic interferon response including interferon-inducible antimicrobial genes as part of the cell-mediated immune response to combat the pathogen.

GBPs are members of the IFN-induced GTPase family and all seven GBPs found in humans are localized in a single cluster on chromosome 1 suggesting that they are regulated by the same pathways [40, 41]. GBPs are cytosolic proteins and this intracellular localization is key for their specific role in the host defense against several intracellular pathogens [42, 43]. Six of the seven human GBPs are upregulated in the RR peripheral blood signature as compared to before reaction and non-reactional patients. *GBP1*, *GBP2* and *GBP5* are specific to the RR signature in comparison to the other clinical forms of leprosy. Of the GBP family, only *GBP1* and *GBP5* were upregulated in RR compared to L-lep/MB and T-lep/PBlesions. All GBPs were upregulated in RR in comparison to L-lep/MBlesions. *GBP1* and *GBP5* were previously reported to be upregulated in the blood of RR patients [29]. *GBP1*, *GBP4* and *GBP5* were also identified as upregulated in the peripheral blood of active TB patients in different cohorts [44–46]. These three GBP genes were identified as part of the sixteen genes related to risk signature for development of active TB in patients with latent TB [47].

An integrative genomics approach to study the peripheral blood before, during and after RR revealed a mixture of type I and type II IFN-specific genes in the peripheral blood signature of patients with active RR. The transcription factors related to IFN signaling, *STAT1*, *IFR7*, *IRF3* and *IRF1*, were also enriched in the RR signature. Previously, a type II IFN gene profile was also detected in lesions of RR patients as well as in the lesions of paucibacillary tuberculoid leprosy patients relative to multibacillary lepromatous patients, consistent with the role of IFN-γ in inducing an antimicrobial response in *M*. *leprae* infected macrophages [23]. The presence of the type II IFN-specific gene signature in the blood of active RR patients but not paucibacillary tuberculoid leprosy patients indicates the systemic nature of the immune response in RR. This may be explained by the presence of few lesions in tuberculoid leprosy with rare bacilli such that the immune response is limited, compared with multiple lesions in RR containing some bacilli such that the amount of IFN-γ produced is greater resulting in a systemic response.

IFN-γ is the strongest upstream regulator identified in the RR peripheral blood transcriptome in this study. Previously the IFN-γ signaling pathway was identified in peripheral blood of RR patients in comparison to non-reactional patients [28, 29]. We found that IFN-γ induced six of the GBP family members, but only *GBP1*, *GBP2* and *GBP5* were more strongly induced by IFN-γ vs. IFN-β; these are the same GBP family members specifically upregulated in RR. Our results are consistent with reports demonstrating that IFN-γ is a stronger inducer of GBP transcription and activation as compared to the type I IFNs and TLR ligands [48, 49]. The induction of GBPs is key for activation of antimicrobial activity against intracellular bacteria [50] and in diseases such as dysentery, toxoplasmosis and sepsis [51–54]. GBPs can interact with autophagy cargo receptors and with galectin to recognize early endosomes containing bacteria [55, 56]. GBPs increase proteasome activation, leading to the production of the pro-inflammatory cytokines IL-1β and IL-18 in macrophages following LPS activation [57]. This is consistent with the finding that whole blood cells from RR patients express more inflammatory genes, such as *CCL2*, *CCL3*, *IL-1A*, *IL1B* and *IL6* when compared to whole blood cells of patients without RR [39].

The presence of a type I IFN downstream gene response in the blood of active RR patients was in contrast to our findings in skin lesions, as such a response was not present in lesions. One of the most upregulated IFN α/β downstream genes in the RR peripheral blood signature is the interferon stimulated gene 15 (*ISG15*), which encodes a ubiquitin-like protein essential for the host defense against mycobacterial infection [58]. The relative absence of a type I IFN response in RR lesions raises questions with respect to the origin of the type I IFN specific downstream signature in RR peripheral blood transcriptome. RR patients are heterogeneous, sometimes with different lesions in a given patient in various stages of evolution, such that some lesions in patients could still be similar to patients with multibacillary disease which express type I IFN downstream genes. Alternatively, the release of bacterial products from lesions into the blood as part of the naturally occurring cell-mediated immune response or from antibiotic therapy could trigger a type I IFN response in circulating leukocytes. Even though both RR and MB are enriched for the IFN-β downstream signature, each signature includes a different set of genes. The RR IFN-β signature includes ISG15, as described previously [58], and the MB IFN-β signature includes *IL10*, with the encoded protein known to inhibit the IFN-γ-mediated antimicrobial response [23]. ISG15 and IL-10 have opposite roles in the immune response against mycobacterial infection [23, 58].

In contrast to the RR patients that expressed an IFN-γ and IFN-β downstream signature, the blood transcriptomes of multibacillary before reaction (BR) were not enriched for any IFN signature. However, we detected an IFN-β signature similar to that found in L-lep/MB patients in the blood of MB patients that did not develop RR [23]. It remains to be tested whether the absence of specific IFN-β downstream genes in multibacillary lesions is predictive of an increased risk for developing RR.

In summary, this study suggests a connection between the activation of cell-mediated immunity and the antimicrobial response in leprosy. The IFN-γ gene expression signature, activated by the release of *M*. *leprae* ligands and antigens spontaneously or in response to the MDT treatment, leads to induction of an antimicrobial response, involving GBP members both systemically at the site of the disease and. This may lead to a further breakdown of bacilli leading to release of ligands and antigens that amplify the immune response, yet also contributes to tissue injury. The link between cell-mediated immunity and the antimicrobial response identifies potential targets for therapy to intervene in RR patients, perhaps by inhibiting specific antimicrobial pathways that paradoxically contribute to inflammation.

## Supporting information

S1 Table Leprosy patient’s classification table. (DOCX)

S2 Table RR temporal stages classification table. (DOCX)

S3 Table 1017 RR upregulated genes. Excel spreadsheet.
(XLSX)

S4 Table 193 RR downregulated genes. Excel spreadsheet.
(XLSX)

S5 Table qPCR primer sequences. (DOCX)

S1 Fig Flow diagram for analysis of gene expression profiles. (TIF)

S2 Fig Unsupervised analysis. (A) Schematic diagram of the reactional patient temporal stages. (B) Unsupervised principal component analysis (PCA) of 30 RR leprosy whole blood specimens. Coefficient of variance was calculated and the top 500 genes were used to PCA analysis. Ellipsoids represent the 95% confidence interval for sample distribution. Total 2-dimensional PCA mapping represents 32.2% of variance (PC1 = 23.1% and PC2 = 9.1%). (C) Unsupervised hierarchical clustering of RR whole blood specimens. Coefficient of variance was calculated and the top 500 genes were clustered using average Pearson correlation and displayed in a tree, with each terminal leaf representing a blood sample. BR, before reaction; RR, reversal reaction; AT, after treatment.
(TIF)

S3 Fig WGCNA and differential expressed genes common genes. (A) Venn diagrams show the 434 overlapped genes between the 1017 upregulated genes in RR (FC≥1.2, p ≤0.05) and 794 genes present in the RR positively correlated WGCNA module, bisque4. Fold enrichment (FE) = 15.38 and hypergeometric p-value = 2.0 e-376. (B) Venn diagrams show the 25 overlapped genes between the 193 downregulated genes in RR (FC≥1.2; p ≤0.05) and 555 genes present in the RR negatively correlated WGCNA module, magenta4. Fold enrichment (FE) = 5.36 and hypergeometric p-value = 1.0 e-376.
(TIF)

S4 Fig Antimicrobial genes are upregulated genes in RR. (A) IPA antimicrobial network groups and members. (B) Venn diagrams show the 6 overlapped genes between the 113 genes from the cured antimicrobial list and 434 common genes between the upregulated genes in RR and RR positively correlated WGCNA module, bisque4. Fold enrichment (FE) = 10.6 and hypergeometric p-value = 1.0 e-33.
(TIF)

S5 Fig Validation of the distribution of the GBP 1 to 6 mRNA levels by qPCR. (A) Distribution of GBPs 1 to 6 mRNA detection in the whole blood of the 10 RR temporal stages; BR (green), RR (red) and AT (blue). The graph normalized counts for all 10 individual per group. Significance was determined by paired one-tailed ANOVA using GraphPad Prism software and post-hoc (Tukey multiple comparison test). (B) GBPs 1 to 6 correlation of RNAseq normalized counts (NC) and qPCR arbitrary units (AU) for all RR temporal stages (BR = 10, green, RR = 10, red and AT = 10, blue). Pearson correlation was used to calculate r values and two-tailed p-value was calculated for correlation significance. (C) Distribution of GBPs 1 to 6 expression (arbitrary units) in the whole blood for all groups of leprosy patients by qPCR. The graph shows the mean per clinical type. (MB = 8, black; BR = 10, green; RR = 10, red; AT = 10, blue and PB = 8, gray). Significance was determined by one-tailed ANOVA using GraphPad Prism software and post-hoc (Tukey multiple comparison test). (D) GBPs 1 to 6 correlation of RNAseq normalized counts (NC) and qPCR arbitrary units (AU) for all leprosy groups. Pearson correlation was used to calculate r values and the two-tailed p-value was calculated for correlation significance. * P <0.05, **P < 0.01, ***P < 0.001. MB = multibacillary, BR = before reaction, RR = reversal reaction, AT = after treatment and PB = paucibacillary.
(TIF)
