# Supplementary material for: Identification of a systemic interferon-γ inducible antimicrobial gene signature in leprosy patients undergoing reversal reaction
Source: PLoS Negl Trop Dis. 2019 Oct 10;13(10):e0007764. doi: 10.1371/journal.pntd.0007764 (PMC6805014; doi:10.1371/journal.pntd.0007764)
Supplement: S1 Table — (DOCX) [file pntd.0007764.s001.docx]

**Table S1**

| Patient | Classification | Age (years) | Gender | Country | BI |
| --- | --- | --- | --- | --- | --- |
| MB1* | MB | 36 | Male | Bangladesh | 2+ |
| MB2* | MB | 39 | female | Bangladesh | 2+ |
| MB3* | MB | 36 | Male | Bangladesh | 0 |
| MB5* | MB | 30 | Male | Bangladesh | 2+ |
| MB7* | MB | 35 | Male | Brazil | 3.2+ |
| MB8* | MB | 29 | Male | Brazil | 2,42+ |
| MB9* | MB | 42 | female | Brazil | 4,28+ |
| MB10* | MB | 40 | female | Nepal | 0,25+ |
| MB13* | MB | N/A | N/A | Ethiopia | 0 |
| MB14* | MB | 17 | Male | Netherlands | 5+ |
| MB15 | MB | 18 | Male | Bangladesh | 4+ |
| MB16 | MB | 72 | Male | Bangladesh | 4+ |
| MB17 | MB | 24 | Male | Bangladesh | 4+ |
| MB18 | MB | 40 | Male | Bangladesh | 1+ |
| MB19 | MB | 34 | Male | Bangladesh | 4+ |
| MB20 | MB | 35 | Male | Bangladesh | 4+ |
| MB21 | MB | 26 | Male | Bangladesh | 3+ |
| MB22 | MB | 30 | Male | Bangladesh | 2+ |
| PB1 | PB | 55 | Male | Bangladesh | 0 |
| PB2 | PB | 65 | Male | Bangladesh | 0 |
| PB3 | PB | 43 | Male | Bangladesh | 0 |
| PB4 | PB | 23 | Male | Bangladesh | 0 |
| PB5 | PB | 63 | Male | Brazil | 0 |
| PB6 | PB | 46 | Male | Brazil | 0 |
| PB7 | PB | 49 | female | Brazil | 0 |
| PB8 | PB | 20 | female | Brazil | 0 |

* Patients with whole blood was taken at the time of diagnostic, as well during the first reaction and during reaction treatment.

MB- multibacillary leprosy

PB- paucibacillary leprosy

BI-bacillary index
