# Supplementary material for: Identification of a systemic interferon-γ inducible antimicrobial gene signature in leprosy patients undergoing reversal reaction
Source: PLoS Negl Trop Dis. 2019 Oct 10;13(10):e0007764. doi: 10.1371/journal.pntd.0007764 (PMC6805014; doi:10.1371/journal.pntd.0007764)
Supplement: S2 Table — (DOCX) [file pntd.0007764.s002.docx]

**Table S2**

| **Patient** | **Leprosy diagnosis** | **Reaction**  **diagnosis** | **After treatment** |
| --- | --- | --- | --- |
| **MB1** | MB1-BR | MB1-RR | MB1-AT |
| **MB2** | MB2-BR | MB2-RR | MB2-AT |
| **MB3** | MB3-BR | MB3-RR | MB3-AT |
| **MB5** | MB5-BR | MB5-RR | MB5-AT |
| **MB7** | MB7-BR | MB7-RR | MB7-AT |
| **MB8** | MB8-BR | MB8-RR | MB8-AT |
| **MB9** | MB9-BR | MB9-RR | MB9-AT |
| **MB10** | MB10-BR | MB10-RR | MB-10-AT |
| **MB13** | MB13-BR | MB13-RR | MB13-AT |
| **MB14** | MB14-BR | MB14-RR | MB14-AT |

MB- multibacillary leprosy

BR- before reaction (at the time of leprosy diagnosis)

RR- reversal reaction (at the time of RR diagnosis)

AT- after treatment (after prednisone treatment)
