# Supplementary material for: Identification of a systemic interferon-γ inducible antimicrobial gene signature in leprosy patients undergoing reversal reaction
Source: PLoS Negl Trop Dis. 2019 Oct 10;13(10):e0007764. doi: 10.1371/journal.pntd.0007764 (PMC6805014; doi:10.1371/journal.pntd.0007764)
Supplement: S5 Table — (DOCX) [file pntd.0007764.s005.docx]

**Table S2**

| **Gene** | **NCBI Reference Sequence** | **Product length (bp)** | **Primer sequence 3→5treatment** |
| --- | --- | --- | --- |
| **h36B4/(RPLP0)** | NM_001002 | 67 | F- CCACGCTGCTGAACATGCT  R- TCGAACACCTGCTGGATGAC |
| **hGBP1** | NM_002053.3 | 189 | F- AAAGAACAGACAAGGGAACAGCC  R- AGGTAGGATTTGCCTGTGCG |
| **hGBP2** | NM_004120.5 | 168 | F- GATTGGCCCGCTCCTAAGAA  R- CTCTAGACGAGGCCCATTGAC |
| **hGBP3** | NM_018284.3 | 194 | F- GATCCACATGACAGGCCCAA  R- AGCCCAGAGAGAAGCCCTTA |
| **hGBP4** | NM_052941.5 | 154 | F- CCCACACCAGGTTATCCAGAA  R- ATAGCCCTACAATGGCCACC |
| **hGBP5** | NM_052942.3 | 269 | F- GCCTCCGCTGCATACAAATC  R- GCAACAGAGAAGCCCTTGTTC |
| **hGBP6** | NM_198460.2 | 168 | F- AGAGCAGGCTCTTCTAGGTT  R- CAGTCCTACAATGGCCACCA |

F- forward primer

R- reverse primer

h- human
