# Supplementary figures and images for: Identification of a systemic interferon-γ inducible antimicrobial gene signature in leprosy patients undergoing reversal reaction
Source: PLoS Negl Trop Dis. 2019 Oct 10;13(10):e0007764. doi: 10.1371/journal.pntd.0007764 (PMC6805014; doi:10.1371/journal.pntd.0007764)

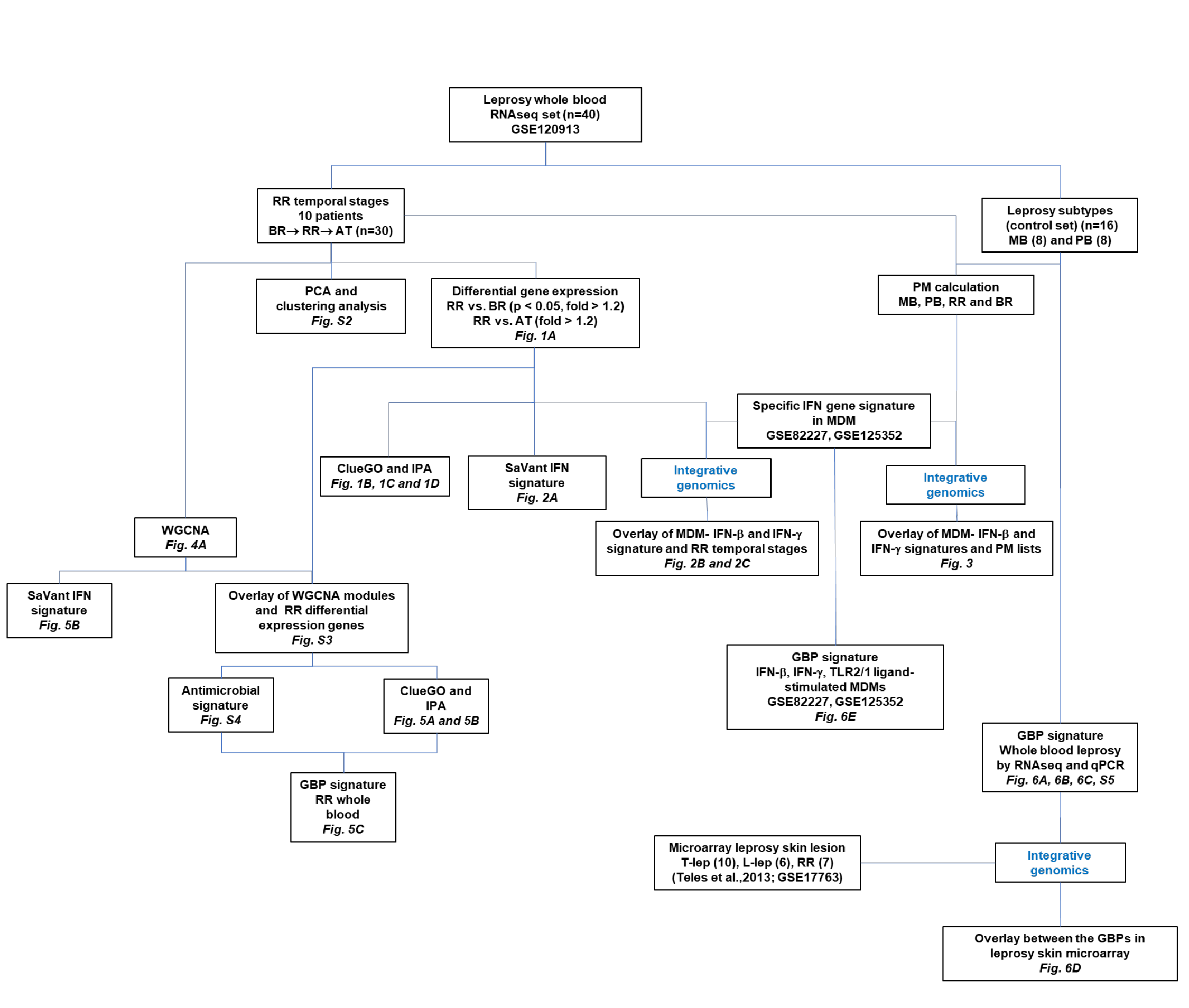

Supplement: S1 Fig — (TIF) [file pntd.0007764.s006.tif]

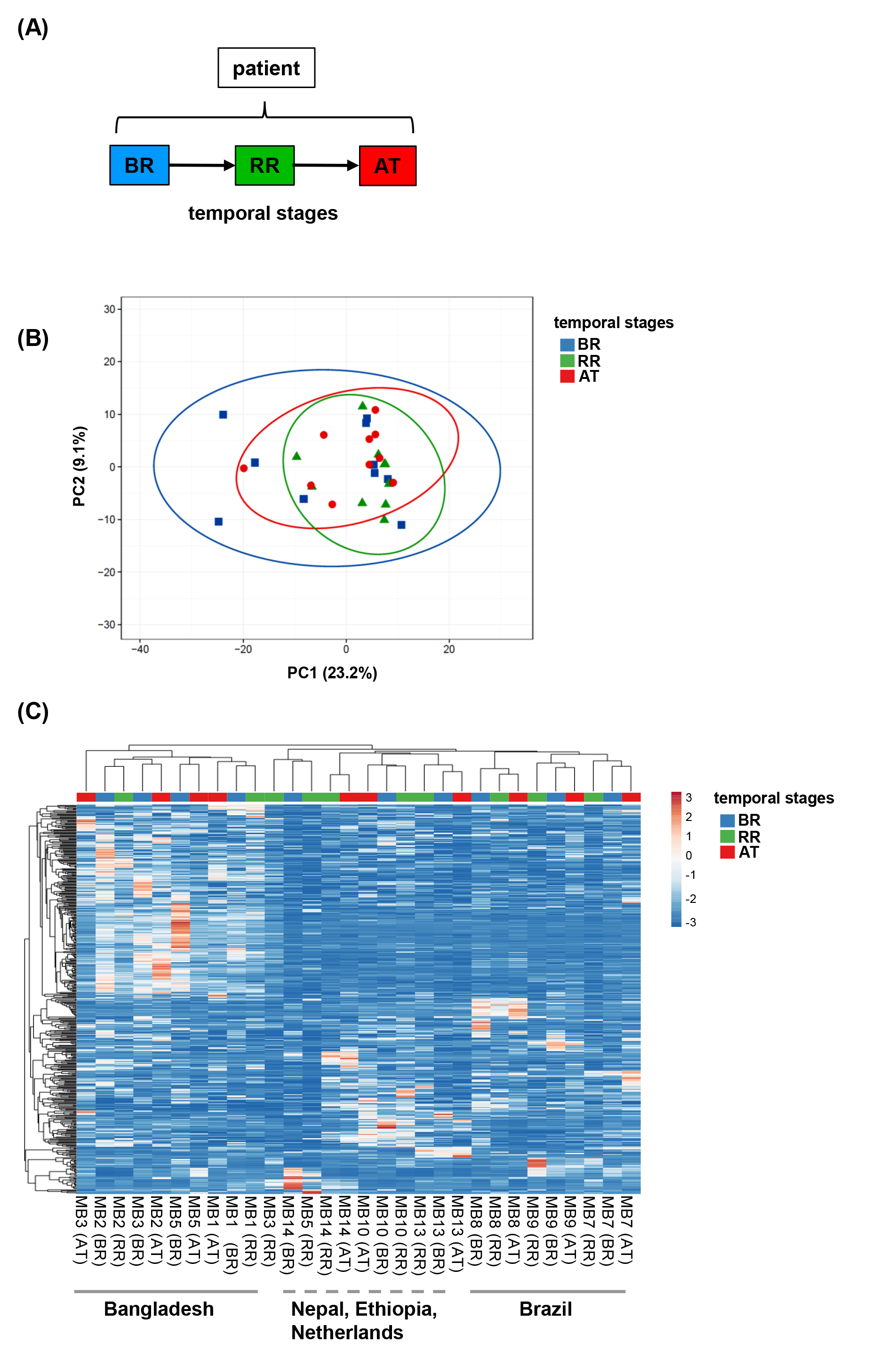

Supplement: S2 Fig — (A) Schematic diagram of the reactional patient temporal stages. (B) Unsupervised principal component analysis (PCA) of 30 RR leprosy whole blood specimens. Coefficient of variance was calculated and the top 500 genes were used to PCA analysis. Ellipsoids represent the 95% confidence interval for sample distribution. Total 2-dimensional PCA mapping represents 32.2% of variance (PC1 = 23.1% and PC2 = 9.1%). (C) Unsupervised hierarchical clustering of RR whole blood specimens. Coefficient of variance was calculated and the top 500 genes were clustered using average Pearson correlation and displayed in a tree, with each terminal leaf representing a blood sample. BR, before reaction; RR, reversal reaction; AT, after treatment. (TIF) [file pntd.0007764.s007.tif]

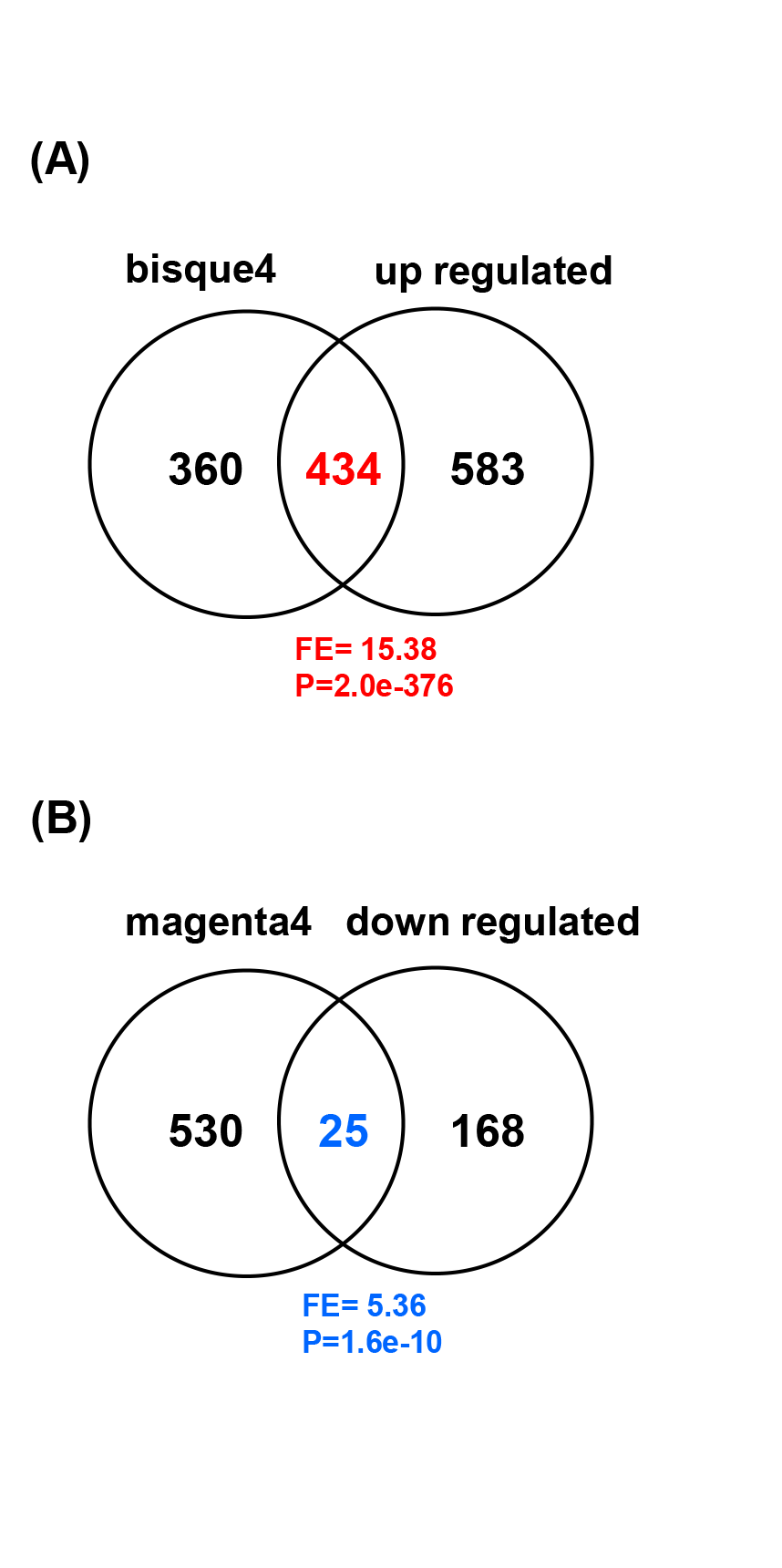

Supplement: S3 Fig — (A) Venn diagrams show the 434 overlapped genes between the 1017 upregulated genes in RR (FC≥1.2, p ≤0.05) and 794 genes present in the RR positively correlated WGCNA module, bisque4. Fold enrichment (FE) = 15.38 and hypergeometric p-value = 2.0 e-376. (B) Venn diagrams show the 25 overlapped genes between the 193 downregulated genes in RR (FC≥1.2; p ≤0.05) and 555 genes present in the RR negatively correlated WGCNA module, magenta4. Fold enrichment (FE) = 5.36 and hypergeometric p-value = 1.0 e-376. (TIF) [file pntd.0007764.s008.tif]

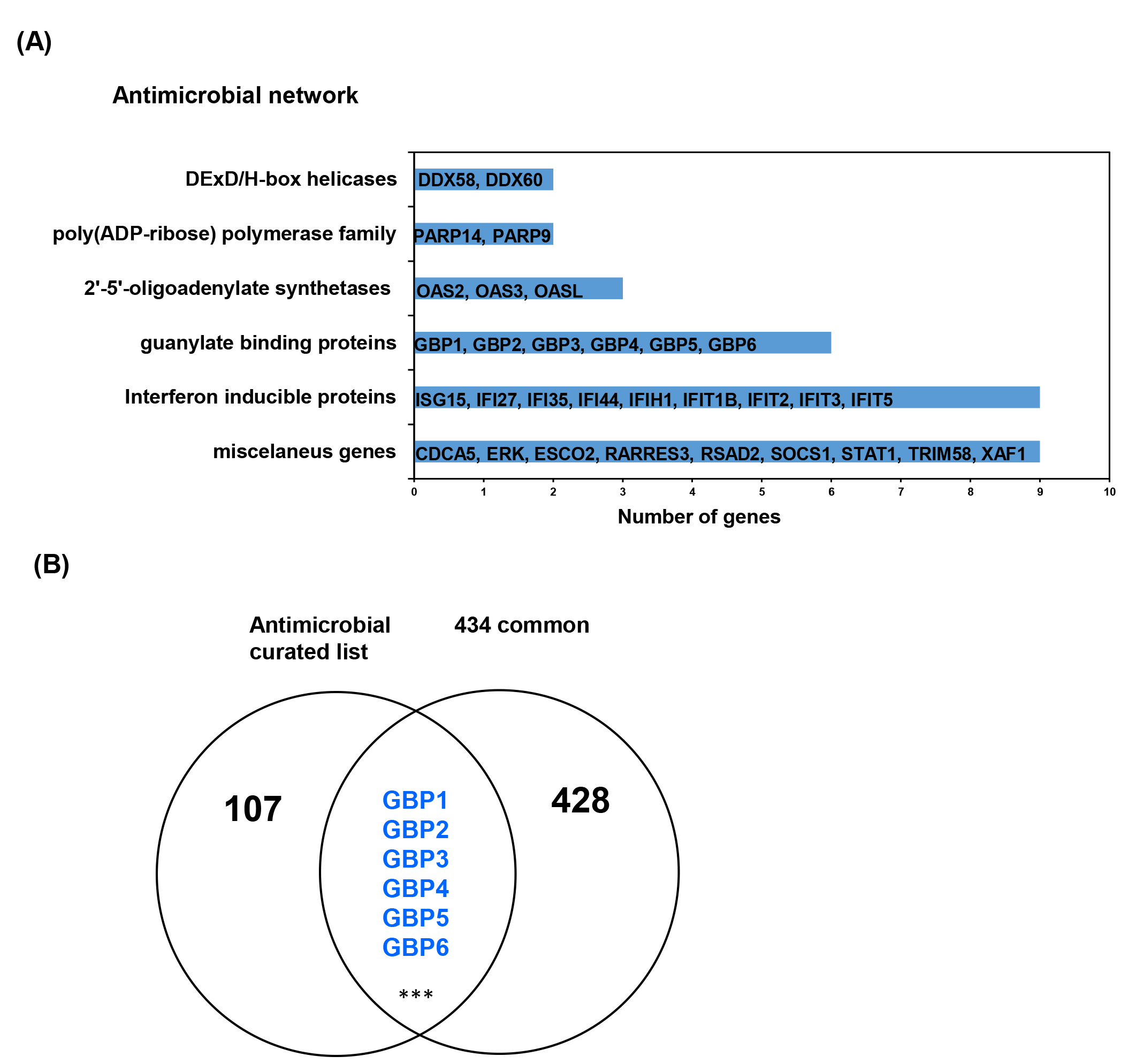

Supplement: S4 Fig — (A) IPA antimicrobial network groups and members. (B) Venn diagrams show the 6 overlapped genes between the 113 genes from the cured antimicrobial list and 434 common genes between the upregulated genes in RR and RR positively correlated WGCNA module, bisque4. Fold enrichment (FE) = 10.6 and hypergeometric p-value = 1.0 e-33. (TIF) [file pntd.0007764.s009.tif]

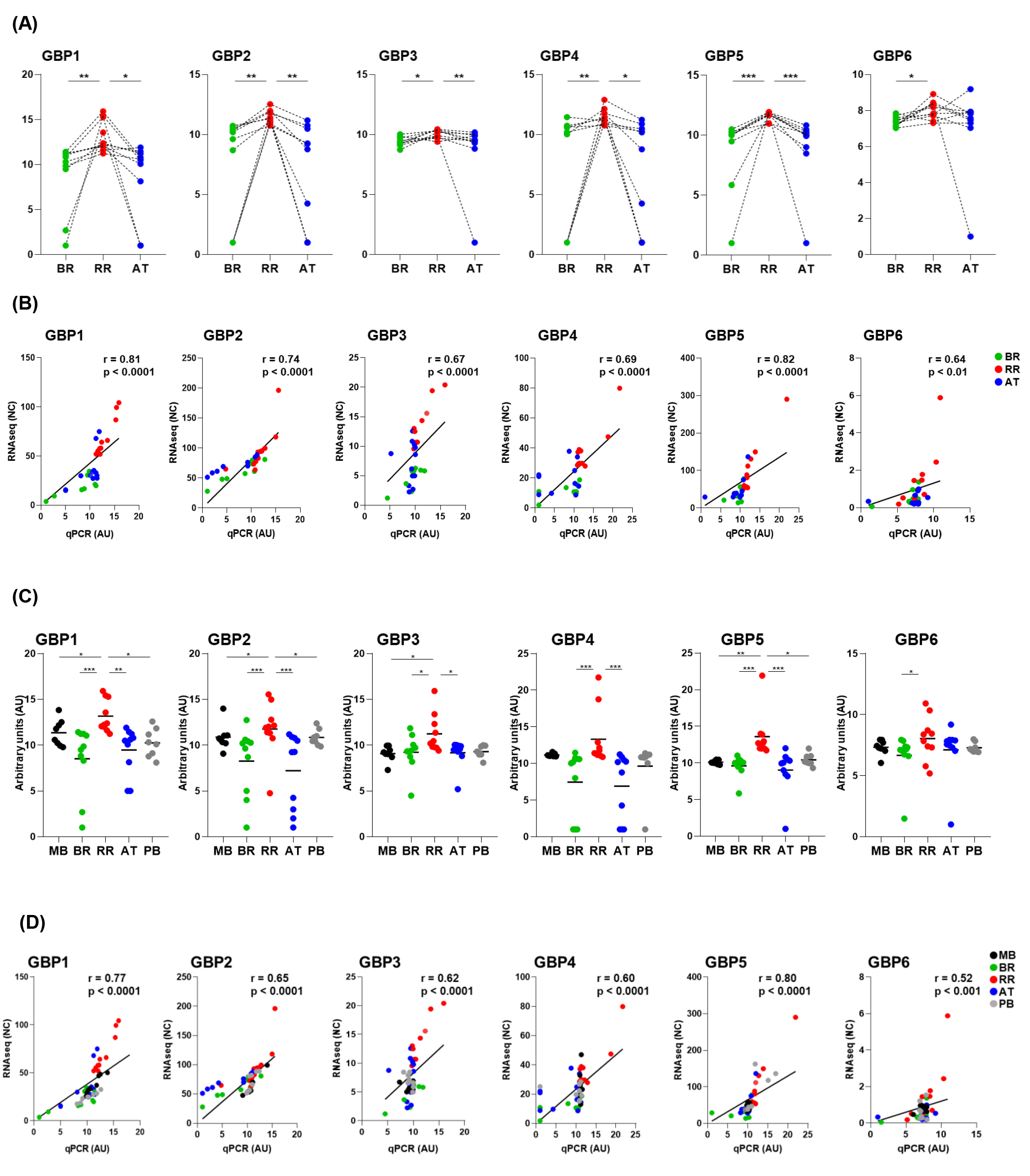

Supplement: S5 Fig — (A) Distribution of GBPs 1 to 6 mRNA detection in the whole blood of the 10 RR temporal stages; BR (green), RR (red) and AT (blue). The graph normalized counts for all 10 individual per group. Significance was determined by paired one-tailed ANOVA using GraphPad Prism software and post-hoc (Tukey multiple comparison test). (B) GBPs 1 to 6 correlation of RNAseq normalized counts (NC) and qPCR arbitrary units (AU) for all RR temporal stages (BR = 10, green, RR = 10, red and AT = 10, blue). Pearson correlation was used to calculate r values and two-tailed p-value was calculated for correlation significance. (C) Distribution of GBPs 1 to 6 expression (arbitrary units) in the whole blood for all groups of leprosy patients by qPCR. The graph shows the mean per clinical type. (MB = 8, black; BR = 10, green; RR = 10, red; AT = 10, blue and PB = 8, gray). Significance was determined by one-tailed ANOVA using GraphPad Prism software and post-hoc (Tukey multiple comparison test). (D) GBPs 1 to 6 correlation of RNAseq normalized counts (NC) and qPCR arbitrary units (AU) for all leprosy groups. Pearson correlation was used to calculate r values and the two-tailed p-value was calculated for correlation significance. * P <0.05, **P < 0.01, ***P < 0.001. MB = multibacillary, BR = before reaction, RR = reversal reaction, AT = after treatment and PB = paucibacillary. (TIF) [file pntd.0007764.s010.tif]
